# Concerns over prognosis prediction for non-small cell lung cancers using major pathological response

**DOI:** 10.1097/JS9.0000000000001032

**Published:** 2023-12-21

**Authors:** Kuo-Chuan Hung, I-Wen Chen, Cheuk-Kwan Sun

**Affiliations:** a Department of Anesthesiology, Chi Mei Medical Center; b Department of Anesthesiology, Chi Mei Medical Center, Liouying, Tainan City; c Department of Emergency Medicine, E-Da Dachang Hospital; d School of Medicine for International Students, College of Medicine, I-Shou University, Kaohsiung City, Taiwan

*Dear Editor,*

We read with interest the systematic review and meta-analysis by Chen *et al*.^1^ that examined the reliability of major pathological response (MPR) as a potential surrogate endpoint for survival in patients with resectable non-small cell lung cancer (NSCLC) receiving neoadjuvant immunotherapy. The authors should be congratulated on this timely and rigorous synthesis of evidence to answer an important clinical question. The study found a pooled MPR rate of 53.8% for neoadjuvant immunotherapy-containing regimens^1^. Critically, MPR was found to be associated with improved disease-free/progression-free/event-free survival (DFS/PFS/EFS) [hazard ratio (HR) 0.28] and overall survival (HR 0.80)^1^, thereby suggesting a positive correlation between MPR and meaningful survival gains with neoadjuvant immunotherapy in patients diagnosed with early NSCLC.

However, DFS/PFS/EFS analysis showed a significant heterogeneity (*I* ^2^=77%)^1^, highlighting the complex nature of NSCLC responses to immunotherapy and the necessity for more nuanced analyses. Thus, additional assessment is needed to confirm the reliability of this relationship before applying it to treatment decisions or trial design. To further evaluate their DFS/PFS/EFS findings despite the high heterogeneity, the use of prediction intervals may be informative. The prediction interval in a meta-analysis is crucial, especially in the presence of high heterogeneity, as it provides a range in which future outcomes are likely to fall^2,3^. Accordingly, we collected the original raw data from that meta-analysis^1^ to calculate the prediction interval for DFS/PFS/EFS using Comprehensive Meta-Analysis (Version 4, Biostat, Englewood, New Jersey, USA). The results showed a wide 95% prediction interval of 0.013–5.982 (Fig. 1), underscoring potential equivocal outcomes that may favor either MPR or non-MPR in future studies. This finding, therefore, highlights the need for further research to confirm the reliability of MPR as a reliable surrogate for DFS/PFS/EFS outcomes.

**Figure 1:**
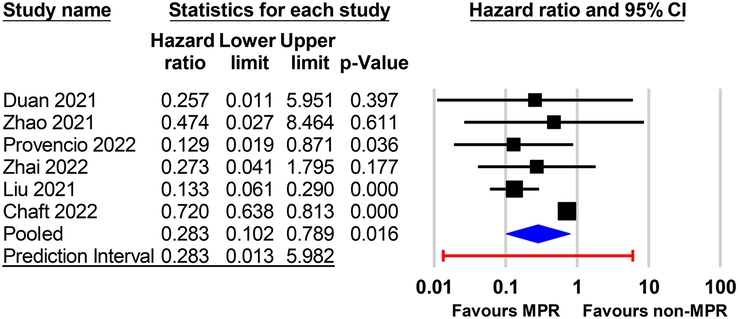
Forest plot displaying individual and pooled hazard ratios with 95% confidence intervals (CIs) and 95% prediction intervals for the association between major pathological response (MPR) and disease-free/progression-free/event-free survival (DFS/PFS/EFS). The diamonds represent the estimated overall effect based on the random effects model. Horizontal lines denote the 95% CI. The 95% prediction interval is indicated by the red line, reflecting the range of effect sizes expected in future studies.

In summary, this study^1^ advances our understanding of the pathological responses and clinical efficacy of neoadjuvant immunotherapy for NSCLC. Although more confirmatory studies are needed, these initial findings suggest the potential usefulness of MPR as a surrogate endpoint to optimize treatment for this patient population. We are grateful to the authors for this timely contribution, which lays the groundwork for future research.

## Ethical approval

Not applicable.

## Consent

Not applicable.

## Sources of funding

Not applicable.

## Author contribution

K.-C.H. and C.-K.S.: wrote the main manuscript text; I-W.C.: prepared Figure 1. All authors read and approved the final version of the manuscript.

## Conflicts of interest disclosure

The authors declare no conflicts of interest.

## Research registration unique identifying number (UIN)

Not applicable.

## Guarantor

Kuo-Chuan Hung.

## Data availability statement

The datasets used and/or analyzed in the current study are available from the corresponding author upon reasonable request.

## Provenance and peer review

This paper was not invited.
